# Comparative analysis of four nutritional scores in predicting hospital stay duration for EICU Patients with acute pancreatitis

**DOI:** 10.3389/fnut.2026.1791814

**Published:** 2026-06-02

**Authors:** Chenpeng Zheng, Guoliang Chen, Chaote Zhang, Ran Zhang, Bin Wu, Xiong Lei

**Affiliations:** 1 Department of Emergency, The First Affiliated Hospital of Wenzhou Medical University, Wenzhou, China; 2 Department of Spine Surgery, The First Affiliated Hospital of Wenzhou Medical University, Wenzhou, China

**Keywords:** acute pancreatitis, NRS-2002, nutritional risk, nutritional scores, prognostic value

## Abstract

**Background:**

Nutritional status significantly impacts outcomes in critically ill patients. Nutritional assessment tools such as the prognostic nutritional index (PNI), controlling nutritional status (CONUT) scores, the Nutritional Risk Screening (NRS-2002), and the Triglyceride and Body Weight Index (TCBI) scores might have prognostic significance in acute pancreatitis (AP). This study aims to investigate the association between these admission scores and the length of hospital stay, as well as their predictive value, in AP patients admitted to the Emergency Intensive Care Unit (EICU).

**Methods:**

Clinical data were retrospectively collected from AP patients admitted to the EICU. Four nutritional scores (including the NRS-2002, PNI, TCBI, and CONUT scores) were calculated within 24 h of admission. Patients were divided into quartiles based on total hospital stay. Linear regression analyses were used to evaluate the associations, and ROC analyses were performed to assess predictive performance. Subgroup analyses were also conducted.

**Results:**

A total of 147 patients were included and divided into quartiles (Q1-Q4) based on hospital stay duration. The NRS-2002 score, the PNI, and the CONUT score showed significant differences across the quartiles. After multivariable adjustment, only NRS-2002 remained independently associated with prolonged hospitalization (MD = 2.98, 95% CI: 1.69–4.27, *p* < 0.001). The ROC analysis demonstrated good predictive ability for NRS-2002 (AUC = 0.813). Subgroup analyses confirmed consistent performance across most patient strata.

**Conclusions:**

The NRS-2002 is the most robust predictor of hospital stay duration among the four nutritional scores evaluated in EICU patients with acute pancreatitis. Routine NRS-2002 screening at admission may help identify high-risk patients for early nutritional intervention.

## 1 Introduction

Acute pancreatitis (AP) is a common gastrointestinal emergency that can range from mild and self-limiting to severe pancreatic necrosis and multiorgan failure. Severe cases often require EICU admission because the condition can deteriorate rapidly (1). These patients tend to have prolonged hospital stays, which not only drive up healthcare costs and use up resources but also increase the risk of complications like secondary infections and organ failure (2). In addition, the inflammatory and hypermetabolic state in AP rapidly depletes nutritional reserves, further hindering recovery (3).

Nutritional assessment and intervention in intensive care unit patients represent a major focus of clinical research. Acute pancreatitis induces severe inflammation and nutritional disturbances, with up to 80% of critical cases experiencing significant protein loss (4). It's well recognized that nutritional status strongly influences clinical outcomes in critically ill patients, including those with pancreatitis (5). Malnutrition weakens immune function, slows tissue repair, and worsens systemic inflammation, all of which can extend hospital stays (6). That's why getting an early and accurate nutritional risk assessment at admission is so important. It helps spot high-risk patients and guide timely intervention, particularly for those admitted to the EICU.

Several tools assess nutritional risk and help identify patients at risk for poor outcomes, including the Nutritional Risk Screening 2002 (NRS-2002), the Prognostic Nutritional Index (PNI), the Triglyceride and Body Weight Index (TCBI), and the Controlling Nutritional Status (CONUT) score. They have been validated in various clinical settings (7, 8). Recent studies suggest these indicators may help predict outcomes in pancreatitis patients (9). However, it is still unclear which one is best at predicting hospital stay length specifically for emergency intensive care unit (EICU) patients with pancreatitis.

Therefore, this study aimed to evaluate and compare the predictive performance of these four nutritional scores for length of hospital stay in EICU patients with acute pancreatitis, to identify the most clinically useful tool for early risk stratification and nutritional intervention.

## 2 Materials and methods

### 2.1 Study population

This retrospective cohort study included 147 adult patients with acute pancreatitis who were admitted to the EICU of the First Affiliated Hospital of Wenzhou Medical University between January 2018 and December 2025. The First Affiliated Hospital Ethics Committee of Wenzhou Medical University approved the research protocol.

### 2.2 Inclusion criteria

Diagnosis of acute pancreatitis followed the revised Atlanta criteria, requiring at least two of: characteristic abdominal pain, serum amylase/lipase>3 times the upper limit normal, or imaging consistent with AP (10).

### 2.3 Exclusion criteria

Patients with acute pancreatitis meeting any of the following criteria were excluded: (1) history of abdominal surgery within 7 days before admission; (2) patients with known chronic pancreatitis or signs of chronic pancreatitis in CT scans; (3) AP caused by periampullary tumor, pre-malignant, and malignant conditions; (4) missing critical data.

### 2.4 Clinical data

Clinical data included demographic characteristics, comorbidities, etiology, laboratory tests within 24 h of admission, total hospital days, and severity scores including APACHE II (Acute Physiology and Chronic Health Evaluation II) and SOFA (Sequential Organ Failure Assessment). Nutritional assessment scores [including NRS-2002 (11), PNI (12), TCBI (13), and CONUT scores (14)]. The calculation formulas for these nutritional scores are as follows: NRS-2002 incorporates the patient's reduced ability to eat, percentage of weight loss in the past 3 months, current body mass index (BMI), age, and comorbidities. PNI = 10 × serum albumin (g/dL) +5 × total lymphocyte count (10^9^/*L*) (if albumin is measured in g/L, divide by 10 to convert to g/dL); TCBI = serum triglycerides (mg/dL) × total cholesterol (mg/dL) × body weight (kg)/1000; CONUT: based on albumin, total cholesterol, and lymphocyte count (14).

### 2.5 Study outcomes

The primary endpoint of this study was the total hospital length of stay for AP patients in the EICU. Patients were categorized into groups according to the quartiles (Q1-Q4) of the length of stay.

### 2.6 Statistical analysis

All statistical analyses were conducted using StataMP 17. Continuous variables were expressed as mean ± SD or median with interquartile range based on normality tested by the Shapiro-Wilk test, while categorical variables were presented as numbers and percentages. Group comparisons utilized *t*-tests, Mann-Whitney U tests, and Fisher's exact test as appropriate.

The study visualized nutritional score distributions using box plots. Linear regression models were employed to evaluate the associations between nutritional scores and hospital stay, with results reported as mean differences (MD) and 95% confidence intervals (CI). The advantage of the multivariate stepwise linear regression model lies in its ability to identify features that make an outstanding contribution, enhance model accuracy, and streamline interpretation. Three models were constructed: an unadjusted model, a partially adjusted model, and a fully adjusted model. Covariates were selected based on both statistical significance in univariate analysis and clinical importance. Subsequently, the predictive performance of the four nutritional scores for prolonged hospital stay was evaluated using receiver operating characteristic (ROC) curve analysis. Finally, to assess the heterogeneity of the association between nutritional scores and hospital stay across different patient subgroups, and to test for interactions, we performed subgroup analyses using adjusted multivariate linear regression models. A *P*-value < 0.05 was considered statistically significant.

## 3 Results

### 3.1 Baseline characteristics

This study included 147 patients with acute pancreatitis from the EICU. They were divided into four groups according to quartiles of hospital stay (Q1 to Q4). The comparison of baseline characteristics across the four groups showed clear differences. Patients in the higher quartiles (longer hospital stay) were generally older (*p* = 0.020) and had lower body weight (*p* = 0.007). They also had higher levels of liver enzymes ALT (*p* = 0.012) and AST (*p* < 0.001). Conversely, they had lower levels of calcium (*p* < 0.001) and albumin (*p* = 0.001). The NRS-2002 score, the PNI, and the CONUT score showed significant differences across the quartiles. Longer hospital stays were linked to lower PNI scores (*p* < 0.001), while NRS-2002 and CONUT scores were significantly higher (both *p* < 0.001) (Table 1).

**Table 1:** Baseline characteristics of participants by quartiles of hospital stay.

–	*N* = 147	Q1 (*N* = 37)	Q2 (*N* = 41)	Q3 (*N* = 35)	Q4 (*N* = 34)	*P* value
Age (year)	44.00 (35.00, 56.00)	42.00 (35.00, 58.00)	39.00 (32.00, 46.00)	47.00 (39.00, 59.00)	50.50 (41.00, 66.00)	0.020
Gender						
Female	50 (34%)	16 (43%)	8 (20%)	9 (26%)	17 (50%)	0.017
Male	97 (66%)	24 (57%)	33 (80%)	26 (74%)	17 (50%)	
BMI	25.91 ± 4.10	25.77 ± 4.47	27.26 ± 4.09	24.87 ± 3.82	25.50 ± 3.69	0.070
Height (cm)	165.86 ± 7.47	165.02 ± 8.13	168.73 ± 8.16	165.68 ± 7.04	163.47 ± 5.08	0.017
Weight (kg)	71.64 ± 14.24	70.67 ± 15.80	77.98 ± 14.74	68.38 ± 11.93	68.39 ± 11.86	0.007
Smoking						
No	74 (50%)	20 (54%)	17 (41%)	14 (40%)	23 (68%)	0.070
Yes	73 (50%)	17 (46%)	24 (59%)	21 (60%)	11 (32%)	
Drinking						
No	77 (52%)	25 (68%)	13 (32%)	15 (43%)	24 (71%)	0.001
Yes	70 (48%)	12 (32%)	28 (68%)	20 (57%)	10 (29%)	
Hypertension						
No	104 (70%)	25 (68%)	30 (73%)	11 (27%)	24 (71%)	0.830
Yes	43 (30%)	12 (32%)	11 (27%)	8 (23%)	10 (29%)	
Diabetes						
No	112 (76%)	23 (62%)	35 (85%)	27 (77%)	27 (79%)	0.110
Yes	35 (24%)	14 (38%)	6 (15%)	8 (23%)	7 (21%)	
Hyperlipemia						
No	106 (72%)	24 (65%)	33 (80%)	24 (69%)	23 (68%)	0.430
Yes	41 (28%)	13 (35%)	8 (20%)	11 (31%)	11 (32%)	
Coronary heart disease						
No	143 (97%)	37 (100%)	39 (95%)	33 (94%)	34 (100%)	0.270
Yes	4 (3%)	0 (0%)	2 (5%)	2 (6%)	0 (0%)	
Tumor						
No	142 (97%)	36 (97%)	40 (98%)	32 (91%)	34 (100%)	0.070
Yes	5 (3%)	1 (3%)	1 (2%)	3 (9%)	0 (0%)	
Etiology						
Hypertriglyceridemic	83 (56%)	21 (57%)	26 (63%)	22 (63%)	14 (41%)	0.170
Biliary	39 (27%)	7 (19%)	9 (22%)	8 (23%)	15 (44%)	
Other	25 (17%)	9 (25%)	6 (14%)	5 (14%)	5 (15%)	
White blood cell (10^9^/L)	12.31 ± 4.98	12.02 ± 5.19	12.65 ± 5.20	11.84 ± 4.85	12.65 ± 4.75	0.860
Red blood cell (10^9^/L)	4.27 (3.73, 5.02)	4.00 (3.41, 4.85)	4.58 (4.04, 5.04)	4.20 (3.46, 4.72)	4.80 (3.85, 5.26)	0.017
Platelet (10^9^/L)	195.00 (144.00, 245.00)	196.00 (143.00, 246.00)	200.00 (149.00, 228.00)	188.00 (107.00, 243.00)	209.50 (144.00, 249.00)	0.780
Hemoglobin (g/dL)	133.95 ± 29.44	141.29 ± 25.43	132.29 ± 28.99	132.29 ± 28.99	135.06 ± 31.34	0.160
Alanine aminotransferase (U/L)	23.00 (15.00, 44.00)	21.00 (13.50, 32.00)	21.00 (13.00, 35.50)	22.00 (15.00, 38.00)	44.50 (18.00, 93.00)	0.012
Aspartate aminotransferase (U/L)	33.00 (23.00, 51.00)	24.00 (18.00, 37.00)	29.00 (21.00, 38.00)	34.00 (26.00, 53.00)	50.50 (35.00, 82.00)	< 0.001
Total bilirubin (umol/L)	20.00 (13.00, 29.50)	16.50 (11.00, 24.50)	18.50 (11.00, 30.00)	19.00 (13.00, 34.00)	24.00 (19.00, 38.00)	0.049
Triglycerides (mg/dL)	433.25 (150.62, 1,346.72)	407.56 (152.39, 1,383.93)	464.26 (162.14, 1,788.83)	373.89 (148.85, 1,346.72)	254.73 (124.93, 810.69)	0.560
Total cholesterol (mg/dL)	206.27 (135.45, 343.66)	207.43 (174.92, 380.81)	229.10 (176.86, 348.69)	154.03 (113.39, 342.50)	183.24 (133.90, 285.61)	0.076
Creatinine (mg/dL)	66.00 (52.00, 99.00)	62.00 (46.00, 86.00)	63.00 (47.00, 92.00)	81.00 (61.00, 139.00)	78.50 (57.00, 112.00)	0.046
Blood glucose (mmol/L)	12.60 (9.30, 16.50)	11.00 (8.50, 14.60)	12.00 (9.20, 16.50)	14.30 (10.60, 18.20)	12.50 (9.70, 15.90)	0.110
Calcium (mg/dL)	1.80 ± 0.30	1.94 ± 0.20	1.87 ± 0.25	1.67 ± 0.30	1.71 ± 0.35	< 0.001
C-reactive protein (mg/L)	239.86 ± 107.83	214.62 ± 106.47	250.94 ± 108.13	270.12 ± 106.20	222.78 ± 105.56	0.110
Amylase (U/L)	204.00 (100.00, 605.00)	138.00 (80.00, 331.00)	171.00 (91.00, 586.00)	173.00 (70.00, 365.00)	598.00 (289.00, 734.00)	0.001
Albumin (g/L)	31.04 ± 4.60	32.18 ± 4.99	32.44 ± 4.30	28.80 ± 4.15	30.44 ± 4.10	0.001
APACHE II	10.00 (8.00, 13.00)	10.00 (7.00, 12.00)	9.00 (7.00, 11.00)	10.00 (7.00, 13.00)	13.00 (10.00, 14.00)	< 0.001
SOFA	2.00 (1.00, 4.00)	1.00 (1.00, 3.00)	2.00 (1.00, 4.00)	3.00 (1.00, 5.00)	2.00 (1.00, 4.00)	0.041
TCBI	6,947.87 (1,355.95, 29,083.09)	5,453.16 (1,962.72, 31,505.59)	11,351.56 (2,148.33, 59,914.04)	4,355.48 (1,129.62, 29,083.09)	3,302.20 (1,098.32, 18,805.14)	0.057
PNI	35.84 ± 5.21	37.30 ± 5.23	37.48 ± 4.80	33.45 ± 5.24	34.75 ± 4.60	< 0.001
NRS-2002	3.38 ± 1.18	2.89 ± 0.81	2.95 ± 1.16	3.68 ± 1.07	4.12 ± 1.23	< 0.001
CONUT	5.52 ± 2.50	4.84 ± 2.48	4.73 ± 2.03	6.71 ± 2.78	6.00 ± 2.23	< 0.001

NRS-2002, Nutritional Risk Screening 2002; PNI, Prognostic Nutritional Index; TCBI, Triglyceride and Body Weight Index; CONUT, Controlling Nutritional Status. Q1, the lowest hospital stay duration group; Q2, the lower hospital stay duration group; Q3, the higher hospital stay duration group. Q4, the highest hospital stay duration group.

### 3.2 Density distributions of four nutritional scores in the short and long hospitalization groups

**Figure 1:**
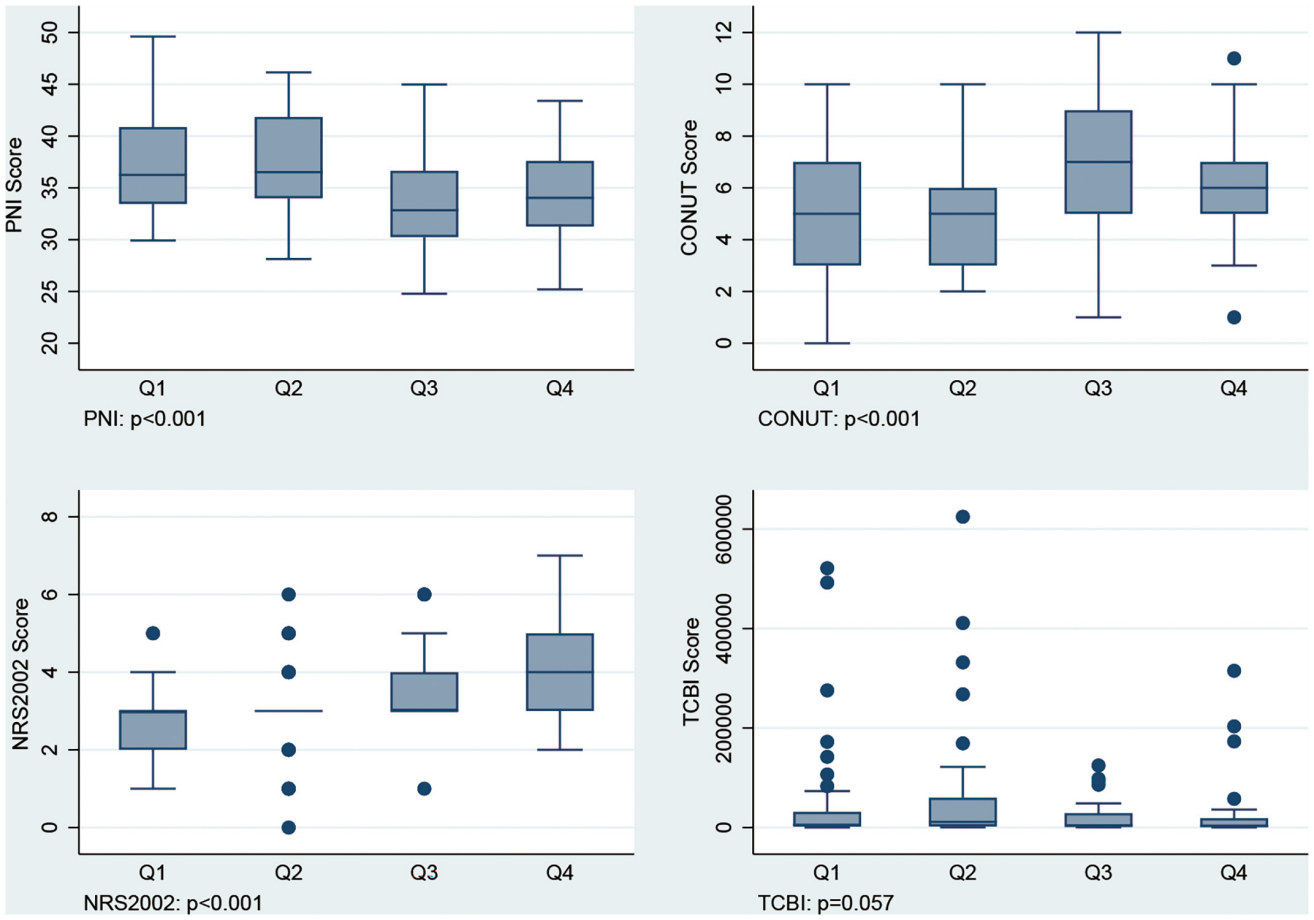
Distribution of four nutritional scores across quartiles of hospital stay.

Patients with longer hospital stays had significantly higher nutritional risk scores (CONUT and NRS-2002), while PNI scores were significantly lower from Q1 to Q4 (Figure 1).

### 3.3 The relationship between four nutritional scores and length of hospital stays

The study used linear regression to test the relationship between four nutritional scores and length of hospital stays. First, without adjustments, higher CONUT and NRS-2002 scores were positively correlated with the length of hospital stay among patients with acute pancreatitis. After adjusting for other key factors, NRS-2002 remained a significant independent predictor. For each point increase in NRS-2002, the hospital stay increased by 2.98 days (95% CI: 1.69 to 4.27, *p* < 0.001). However, after full adjustment, CONUT (*p* = 0.192) and PNI (*p* = 0.105) were no longer significantly associated with hospital stay (Table 2).

**Table 2:** The relationship between four nutritional scores and length of hospital stays.

–	Model 1		Model 2		Model 3	
	MD (95% CI)	*P* value	MD (95% CI)	*P* value	MD (95% CI)	*P* value
TCBI	−0.00 (−0.00 to 0.00)	0.114	excluded	–	excluded	–
CONUT	1.32 (0.723 to 1.916)	< 0.001	1.17 (0.46 to 1.88)	0.001	0.68 (−0.35 to 1.71)	0.192
PNI	−0.55(−0.84 to −0.26)	< 0.001	−0.46 (−0.80 to −0.12)	0.008	−0.59 (−1.31 to 0.12)	0.105
NRS-2002	3.65 (2.45 to 4.85)	< 0.001	3.43 (2.11 to 4.75)	< 0.001	2.98 (1.69 to 4.27)	< 0.001

OR, odds ratio; CI, confidence intervals. The model 1 was not adjusted for any covariates. The model 2 was adjusted by Sex, Age, Hypertension, Diabetes, Hyperlipidemia, Coronary heart disease, Tumor, BMI, Smoking, Drinking. The model 3 was adjusted by Sex, Age, Hypertension, Diabetes, Hyperlipidemia, Coronary heart disease, Tumor, BMI, Smoking, Drinking, Etiology, PACHE II, SOFA, Aspartate Aminotransferase, Creatinine, Calcium, Albumin, Amylase.

When patients were stratified by quartiles of hospital stay, a clear dose-response pattern emerged for NRS-2002. In the fully adjusted model (Model 3), compared with the Q1 group (shortest hospital stay), the NRS-2002 score was progressively higher across quartiles, with MD values of 1.55 (95% CI: 0.38 to 2.71, *p* = 0.010) for Q3 and 5.03 (95% CI: 2.44 to 7.63, *p* < 0.001) for Q4. Similarly, CONUT and PNI also showed significant associations in the unadjusted and partially adjusted models; however, these associations were attenuated and no longer statistically significant after full adjustment. In contrast, TCBI showed no significant association across any of the quartiles or models (Table 3).

**Table 3:** The relationship between four nutritional scores and quartiles of hospital stay.

–	Q1	Q2		Q3		Q4	
		MD (95% CI)	*P* value	MD (95% CI)	*P* value	MD (95% CI)	*P* value
**TCBI**							
Model 1	Ref.	−0.00 (−0.00 to 0.00)	0.465	−0.00 (−0.00 to 0.00)	0.114	−0.00 (−0.00 to 0.00)	0.274
Model 2	Ref.	−0.00 (−0.00 to 0.00)	0.591	−0.00 (−0.00 to 0.00)	0.356	−0.00 (−0.00 to 0.00)	0.902
Model 3	Ref.	−0.00 (−0.00 to 0.00)	0.447	−0.00 (−0.00 to 0.00)	0.899	−0.00 (−0.00 to 0.00)	0.921
CONUT							
Model 1	Ref.	0.25 (0.02 to 0.49)	0.035	0.75 (0.35 to 1.14)	< 0.001	2.40 (1.21 to 3.59)	< 0.001
Model 2	Ref.	0.32 (0.05 to 0.60)	0.022	0.93 (0.47 to 1.40)	< 0.001	2.24 (0.72 to 3.75)	0.004
Model 3	Ref.	0.04 (−0.40 to 0.49)	0.843	0.27 (−0.50 to 1.04)	0.486	1.94 (−0.04 to 3.92)	0.054
PNI							
Model 1	Ref.	−0.12 (−0.23 to 0.02)	0.024	−0.38 (−0.57 to 0.18)	< 0.001	−0.98 (−1.57 to −0.34)	0.001
Model 2	Ref.	−0.15 (−0.27 to 0.03)	0.015	−0.49 (−0.71 to −0.27)	< 0.001	−0.79 (−1.51 to −0.07)	0.032
Model 3	Ref.	−0.02 (−0.32 to 0.28)	0.902	−0.10 (−0.64 to −0.44)	0.713	−1.36 (−2.82 to −0.10)	0.067
NRS-2002							
Model 1	Ref.	0.20 (−0.33 to 0.74)	0.449	2.22 (1.17 to 3.26)	< 0.001	6.29 (4.07 to 8.51)	< 0.001
Model 2	Ref.	−0.01 (−0.57 to 0.55)	0.971	2.00 (0.82 to 3.19)	0.001	6.34 (3.63 to 9.04)	< 0.001
Model 3	Ref.	−0.09 (−0.52 to 0.70)	0.772	1.55 (0.38 to 2.71)	0.010	5.03 (2.44 to 7.63)	< 0.001

OR, odds ratio; CI, confidence intervals. The model 1 was not adjusted for any covariates. The model 2 was adjusted by Sex, Age, Hypertension, Diabetes, Hyperlipidemia, Coronary heart disease, Tumor, BMI, Smoking, Drinking. The model 3 was adjusted by Sex, Age, Hypertension, Diabetes, Hyperlipidemia, Coronary heart disease, Tumor, BMI, Smoking, Drinking, Etiology, PACHE II, SOFA, Aspartate Aminotransferase, Creatinine, Calcium, Albumin, Amylase.

**Figure 2:**
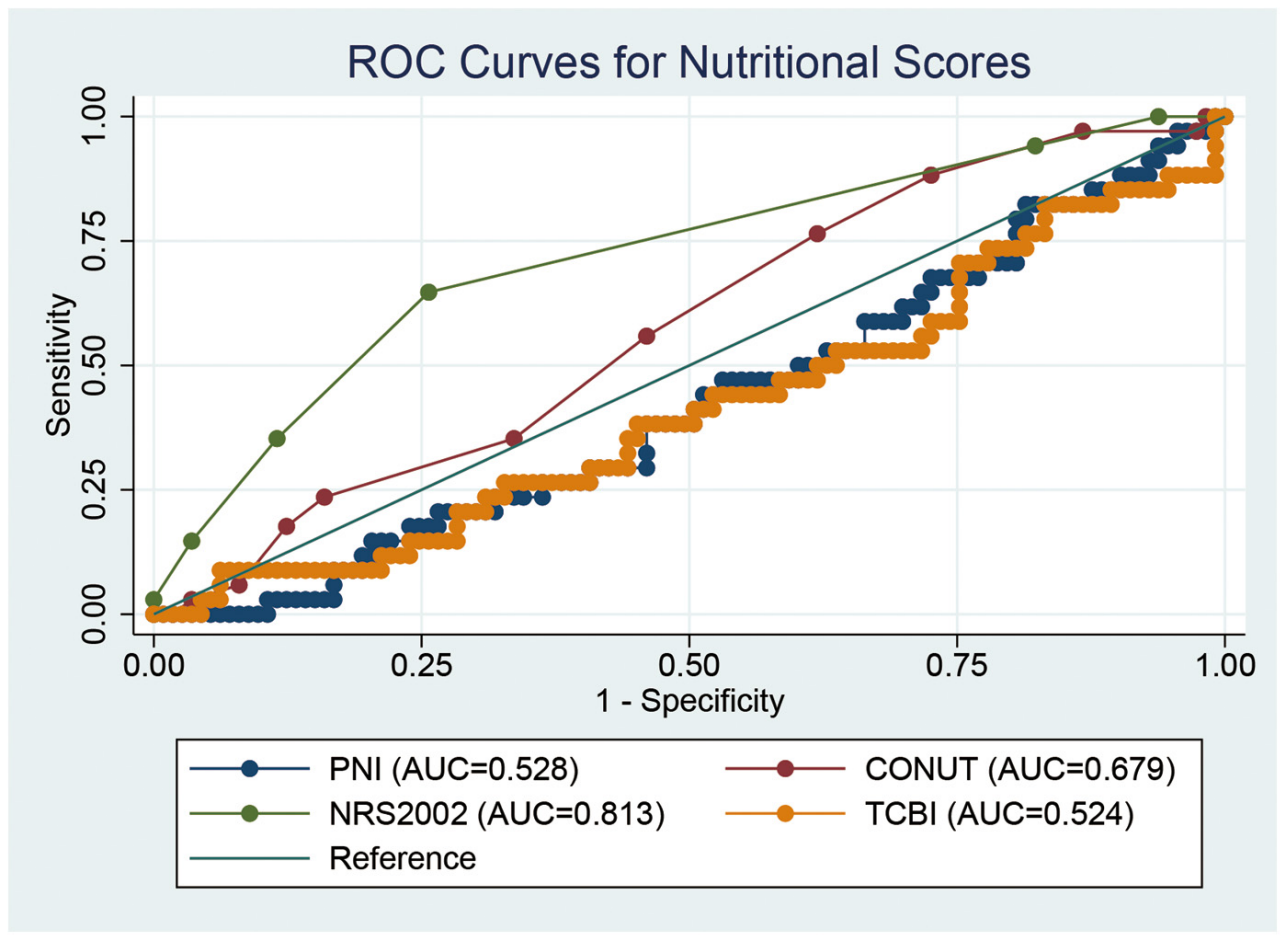
The ROC curve analysis of four nutritional scores.

The study further assessed the predictive performance of the four nutritional scores using ROC curve analysis. The results showed that NRS-2002 had the highest predictive accuracy for prolonged hospital stay, with an area under the curve (AUC) of 0.813. CONUT yielded an AUC of 0.679, indicating moderate predictive performance. In contrast, PNI (AUC = 0.528) and TCBI (AUC = 0.524) showed poor predictive ability (Figure 2).

### 3.4 Subgroup analysis

**Figure 3:**
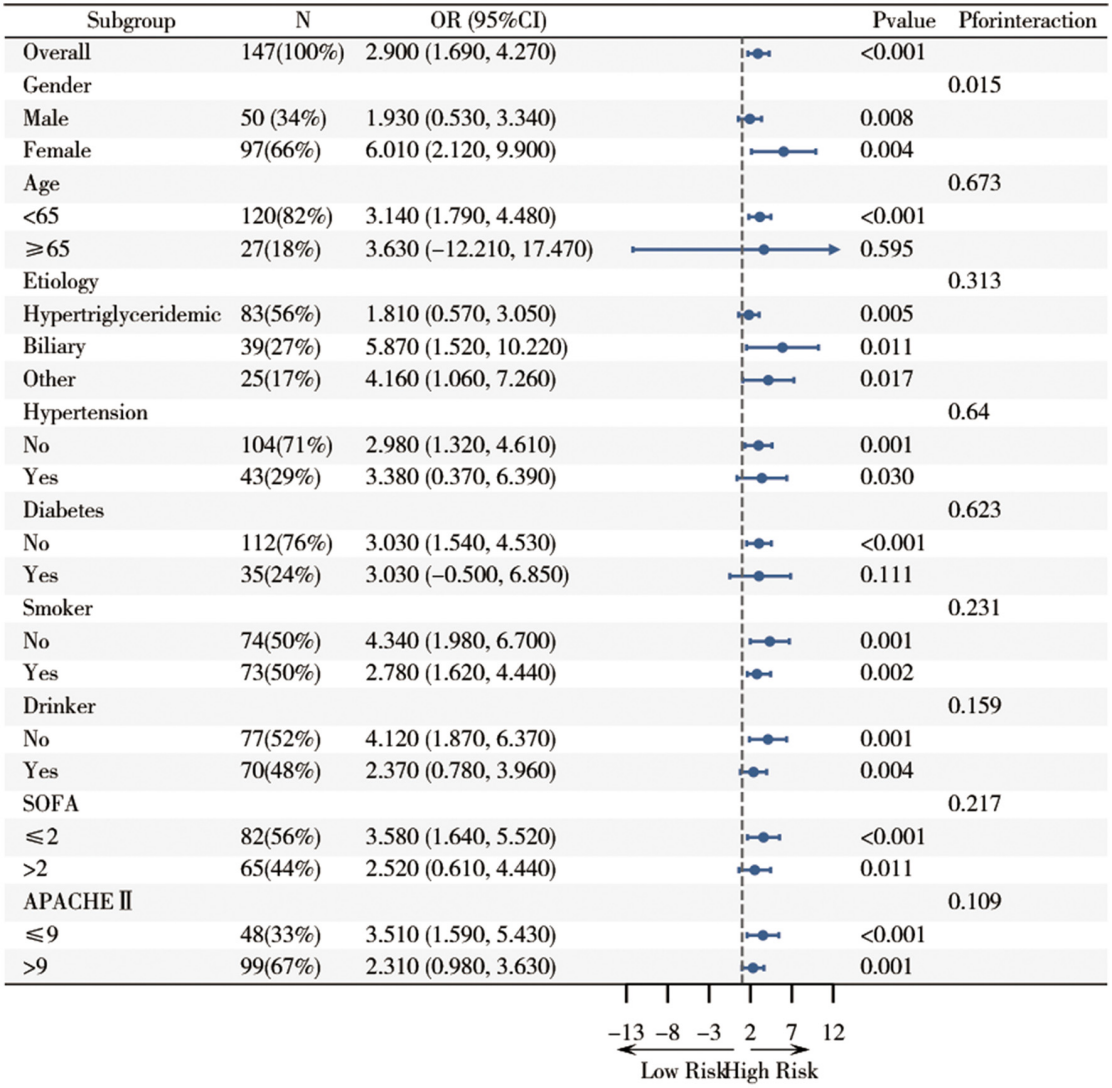
Subgroup analyses for the association of NRS-2002 with hospital stay duration.

The subgroup analyses showed that a higher NRS-2002 score was consistently linked to a longer hospital stay across most patient groups. The association was significant in both male (MD = 1.93, 95% CI: 0.53 to 3.34, *p* = 0.008) and female patients (MD = 6.01, 95% CI: 2.12 to 9.90, *p* = 0.004), with a significant interaction by gender (*p* for interaction = 0.015), indicating a stronger effect in females. The predictive effect held across age groups (< 65 years and ≥65 years), hypertension status, smoking status, drinking status, and SOFA score categories. Importantly, no significant interactions were observed for age, etiology, hypertension, diabetes, smoking, drinking, or SOFA (all p for interaction > 0.05). The association remained significant across all etiological subgroups, including hypertriglyceridemic (MD = 1.81, 95% CI: 0.57 to 3.05, *p* = 0.005), biliary (MD = 5.87, 95% CI: 1.52 to 10.22, *p* = 0.011), and other causes (MD = 4.16, 95% CI: 1.06 to 7.26, *p* = 0.017) (Figure 3).

## 4 Discussion

We tested four nutritional scores (NRS-2002, PNI, TCBI, CONUT) as predictors of EICU hospital stay in acute pancreatitis patients. All were initially associated with stay length. However, after adjusting for other factors, only NRS-2002 remained a strong independent predictor. Its predictive ability was good (AUC = 0.813) and consistent across most patient groups.

While some studies indicate that the CONUT and PNI scores show promise in predicting necrotizing pancreatitis, our research found that these scores did not demonstrate utility in forecasting the length of hospital stay (15). In contrast, the relatively lower predictive value of PNI and CONUT in our cohort may stem from their heavy reliance on laboratory parameters like albumin, which can be rapidly altered by acute-phase responses and fluid resuscitation, thus not solely reflecting baseline nutritional status in the acute setting. The TCBI, while offering a simple assessment of energy reserves, may not fully encompass the complex protein catabolism and systemic inflammation central to pancreatitis pathophysiology.

Additionally, while malnutrition (NRS-2002>3) has been established to impact patient length of stay and healthcare costs, its specific implications in acute pancreatitis patients within the EICU setting remain underexplored (16). The study indicated significantly elevated NRS-2002 in acute pancreatitis patients who required extended EICU stays, relative to their counterparts with shorter hospitalization durations. The integration of novel interdisciplinary knowledge can significantly enhance our in-depth understanding of diseases. Several potential factors may contribute to the risk of malnutrition in acute pancreatitis patients within the EICU. The superior performance of NRS-2002 is likely attributable to its comprehensive and pathophysiology-oriented design. Unlike PNI (primarily reflecting visceral protein status) or CONUT (focused on serum albumin and cholesterol), NRS-2002 incorporates both nutritional status and disease severity. Specifically, it assigns additional points for acute metabolic stress, which is a hallmark of moderate to severe acute pancreatitis. This feature allows NRS-2002 to better capture the synergistic detrimental effects of pre-existing nutritional depletion and the hypercatabolic, inflammatory state induced by pancreatitis itself. Consequently, it more accurately identifies patients whose physiological reserve is insufficient to withstand the illness, leading to protracted recovery and EICU stays. This aligns with previous studies in critical care settings, where NRS-2002 has been validated as a predictor of clinical outcomes for various conditions (17, 18).

It is widely acknowledged that nutritional status can deteriorate during hospitalization. An important contributing factor is the inadequate recognition of this issue by healthcare professionals, which in turn leads to prolonged hospital stays (19). Recent studies have suggested that high protein delivery in critically ill patients may be associated with poorer in-hospital outcomes, including increased mortality (20). The initiation of early nutritional support in EICU patients may be associated with an increased 28-day mortality, according to some studies (21). However, among patients with severe acute pancreatitis, the initiation of enteral nutrition within 48 h is associated with improved clinical outcomes (22). Beyond nutritional management, the refinement of EICU care can not only contribute to symptom alleviation in patients with acute pancreatitis but also reduce the length of hospital stay (23). The comprehensive design of NRS-2002 likely accounts for its superior performance. It integrates nutrition status, disease severity, and age, capturing the complex interplay of factors in critically ill patients. While tools like PNI and CONUT, which are based on lab parameters like albumin, have shown predictive value in other contexts (such as identifying necrotizing pancreatitis or predicting short-term prognosis) (15, 24). They lost independence in our multivariate model. This suggests that in predicting overall hospital stay, their information is already reflected in standard clinical and laboratory data (25, 26). Although the TCBI (based on triglycerides, cholesterol, and weight) performed poorly in our cohort, other metabolic markers show strong links to disease outcomes. A recent meta-analysis confirms the TyG index's association with severity (27), and hyperglycemia during hospitalization is a known risk factor for longer stays (28). This contrast suggests that while metabolic status is important, the characteristic lipid metabolism disruption in pancreatitis may specifically compromise lipid-based tools like TCBI (29).

Subgroup analyses confirmed that NRS-2002 is a robust predictor of hospital stay duration for most patient groups. We observed a significant interaction by gender (*p* for interaction = 0.015), with a stronger association in female patients (MD = 6.01, 95% CI: 2.12–9.90, *p* = 0.004) than in male patients (MD = 1.93, 95% CI: 0.53–3.34, *p* = 0.008). The association remained significant across all etiological subgroups. However, no significant association was found in the diabetic subgroup or among elderly patients (aged ≥65 years), which may be related to the relatively small sample sizes in these subgroups. These findings warrant further validation through large-scale multicentre studies.

Several limitations of this study should be acknowledged. First, it was a single-center retrospective study with a relatively small sample size (*n* = 147), which may limit the generalizability of our findings and reduce statistical power for some subgroup analyses, such as in diabetic patients. Second, we used hospital stay as the primary outcome, but this endpoint is influenced by many factors beyond nutritional status, including institutional practices and discharge policies. We also did not examine other clinically important outcomes, such as in-hospital mortality, organ failure, or pancreatic necrosis. Residual confounding by unmeasured factors cannot be ruled out despite multivariable adjustment. These include nutritional support during hospitalization and etiology-specific interventions like ERCP for biliary AP, plasmapheresis for HTGAP, and drainage for infected necrosis. Finally, the retrospective design means we cannot draw causal conclusions. Large-scale, prospective multicentre studies are needed to validate our findings.

## 5 Conclusion

In conclusion, our findings underscore the critical value of systematic nutritional risk assessment at admission for acute pancreatitis patients. Among the tools evaluated, NRS-2002 emerges as the most pragmatic and effective for early identification of high-risk patients likely to require extended intensive care. Implementing routine NRS-2002 screening could facilitate timely and aggressive nutritional support, potentially mitigating the catabolic state and improving resource allocation in the EICU, especially in acute pancreatitis patients. Further research should explore the impact of nutrition therapy guided by these scores on clinical outcomes.

## Data Availability

The original contributions presented in the study are included in the article/supplementary material, further inquiries can be directed to the corresponding authors.
